# An Enumerative Combinatorics Model for Fragmentation Patterns in RNA Sequencing Provides Insights into Nonuniformity of the Expected Fragment Starting-Point and Coverage Profile

**DOI:** 10.1089/cmb.2016.0096

**Published:** 2017-03-01

**Authors:** Celine Prakash, Arndt Von Haeseler

**Affiliations:** ^1^ Max F. Perutz Laboratories (MFPL), Center for Integrative Bioinformatics Vienna (CIBIV), University of Vienna, Medical University of Vienna, Vienna, Austria.; ^2^ Bioinformatics and Computational Biology, Faculty of Computer Science, University of Vienna, Vienna, Austria.

**Keywords:** enumerative combinatorics, expected starting-point distribution, fragmentation model, RNA sequencing, unbiased coverage

## Abstract

**RNA sequencing (RNA-seq) has emerged as the method of choice for measuring the expression of RNAs in a given cell population. In most RNA-seq technologies, sequencing the full length of RNA molecules requires fragmentation into smaller pieces. Unfortunately, the issue of nonuniform sequencing coverage across a genomic feature has been a concern in RNA-seq and is attributed to biases for certain fragments in RNA-seq library preparation and sequencing. To investigate the expected coverage obtained from fragmentation, we develop a simple fragmentation model that is independent of bias from the experimental method and is not specific to the transcript sequence. Essentially, we enumerate all configurations for maximal placement of a given fragment length, *F*, on transcript length, *T*, to represent every possible fragmentation pattern, from which we compute the expected coverage profile across a transcript. We extend this model to incorporate general empirical attributes such as read length, fragment length distribution, and number of molecules of the transcript. We further introduce the fragment starting-point, fragment coverage, and read coverage profiles. We find that the expected profiles are not uniform and that factors such as fragment length to transcript length ratio, read length to fragment length ratio, fragment length distribution, and number of molecules influence the variability of coverage across a transcript. Finally, we explore a potential application of the model where, with simulations, we show that it is possible to correctly estimate the transcript copy number for any transcript in the RNA-seq experiment.**

## 1. Introduction

**FIG. 1.**
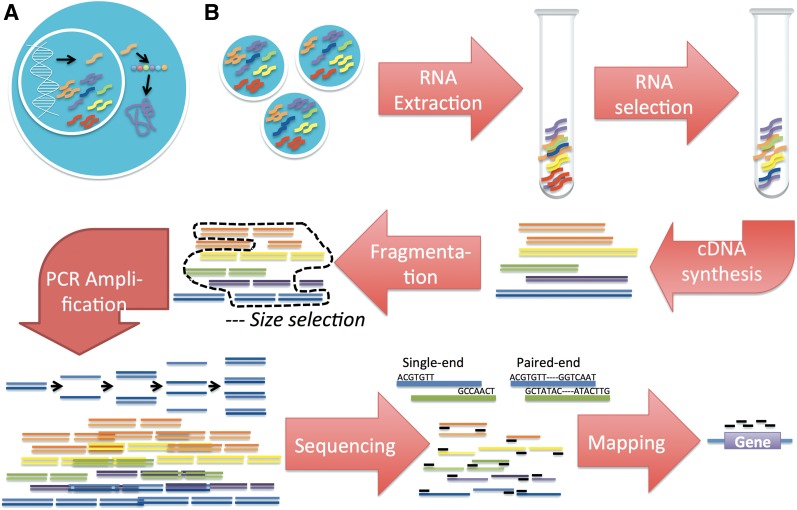
**(A)** The central dogma of molecular biology. Within a cell, a gene is transcribed into RNA that is translated into a chain of amino acids, which fold to form a protein. **(B)** A typical RNA sequencing experiment. RNAs are isolated from cells and the desired population of RNAs is selected. The selected RNAs (of various lengths) are reverse transcribed into double-stranded cDNA. This is followed by fragmentation to an optimal size distribution and may include the step of size selection. cDNA fragments undergo PCR amplification and the amplified products are sequenced to obtain reads that contain information of the series of bases present in the fragment. The cDNA fragment may be sequenced from either one end (single-end sequencing) or from both ends (paired-end sequencing). Data analysis involves mapping the sequenced reads back to the genome and using the aggregate of read counts on a genomic feature to derive expression. PCR, polymerase chain reaction.

In cells, genes are expressed by being transcribed into RNAs, and transcripts may then be translated into proteins (Fig. 1A). RNA sequencing (RNA-seq) is a method to quantify the transcripts expressed in a cell or cell population (Mortazavi et al., 2008; Wang et al., 2009; Djebali et al., 2012; Owens et al., 2016). In an RNA-seq experiment (Fig. 1B), RNAs of interest are isolated and reverse transcribed to a double-stranded complementary DNA (cDNA) such that they can be sequenced (Chu and Corey, 2012). RNAs come in various lengths—in humans, they range from less than 100 nucleotides (Parisien et al., 2013) to greater than 100,000 nucleotides (Bang et al., 2001). Before sequencing, long RNA molecules require fragmentation, typically done prior or subsequent to cDNA conversion (Levin et al., 2010; Head et al., 2014). An optimal fragment length is chosen to maximize the fraction of each fragment sequenced or to match the size of the genomic regions of interest (Head et al., 2014). Fragments in the desired size range can be obtained through fragmentation conditions or with an additional step of size selection (Bronner et al., 2001). The fragmented cDNA molecules are often amplified using polymerase chain reaction (PCR)—where both strands undergo repeated cycles of copying. The amplified products are then sequenced to obtain reads. Reads provide a series of bases in the fragment, from which the identity of the RNA can be derived. Of note, read lengths differ between technologies (Liu et al., 2012). In addition, the cDNA fragment may be sequenced from either one end (single end) or from both ends (paired end) (Sengupta et al., 2011). Mapping reads involves aligning them to the reference genome sequence (Schbath et al., 2012). Read coverage, which is the sum of overlapping reads, provides the extent to which a genomic position is sequenced. In RNA-seq, measures of expression are derived from an aggregate of read counts within a genomic feature (exon, transcript, or gene) (Wilhelm and Landry, 2009). If each position of the genomic feature is sequenced equivalently, coverage of reads across the feature is uniform. Unfortunately, nonuniform coverage has been a perennial concern in RNA-seq (Li et al., 2010; Hower et al., 2012). The lower covering probabilities at positions around a terminus of a sequenced feature, termed “edge effects,” have been explained by Wendl (2006). However, nonuniformity has also been observed in the transcript body. This has been attributed to experimental biases and available methods account and correct for them (Li et al., 2010; Benjamini and Speed, 2012; Hower et al., 2012; Ma and Zhang, 2013). Even though uniformity of coverage is used to measure data quality (DeLuca et al., 2012; Hower et al., 2012; Wang et al., 2012) and correction for nonuniformity or quantification of isoform expression (Jiang and Wong, 2009) is based on assumptions of uniform sampling across the genomic feature, the bias-free expected coverage distribution has yet to be investigated.

**FIG. 2.**
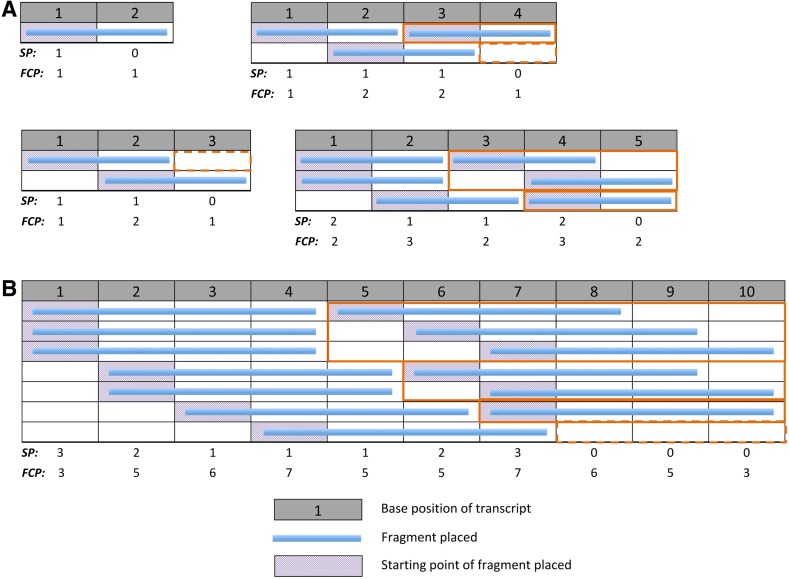
The fragment placement pattern space for various transcript lengths. **(A)** Fragments of 2 bases long placed on transcripts of lengths 2–5 bases. **(B)** Fragments of 4 bases long placed on a transcript of 10 bases long. Each row represents a unique fragmentation pattern where fragments are placed till remaining positions on the transcript permit no further placement of a fragment. The computed starting-point profile, ***SPP***, and fragment coverage profile, ***FCP***, are, respectively, the sum of fragment starting-points (boxes shaded red) and the sum of fragments covering each position, and are shown under each pattern space. Sections that have been bordered orange show pattern spaces of shorter transcripts found in longer transcripts. Dashed orange borders show the pattern spaces for transcripts shorter than the fragment length. ***FCP***, fragment coverage profile.

Recent RNA-seq projects have been directed toward measuring lower amounts of starting RNA (Adiconis et al., 2011; Saliba et al., 2014), where the majority of the fragments from each molecule are sampled and thus fragment starting-points (SPs) (boxes shaded red in Fig. 2) on a transcript are not independent. Here, we suggest a model of unbiased fragmentation of a single transcript in which we enumerate all possible ways to obtain the maximum number of fragments of a desired length from a transcript. Under our model, we produce the expected coverage for varying fragment length to transcript length ratios. To inch closer to reality, we also explore the influence of multiple fragment lengths and its distribution on the coverage profile. We simulate unbiased sequencing experiments that start with a fragmentation process based on our model and investigate the possibility to estimate the number of molecules fragmented in the RNA-seq experiment through SP distributions.

## 2. The Fragmentation Model

### 2.1. Enumerating the fragmentation pattern space for a single transcript

We propose a simple model of fragmentation: A fragmented molecule is represented with fragments of length, *F*, assigned on nonoverlapping positions of a transcript of length, *T* (see Table 1 for notations). Fragments may be assigned with gaps between them but gap lengths must be shorter than a fragment length—that is, we assume that fragmentation of a molecule involves exhaustive fragment placement. We define each unique configuration of exhaustive fragment placement as a *Fragmentation Pattern* and the *Pattern Space* comprises all unique fragmentation patterns possible, given *F* and *T* (some examples shown in Fig. 2).

**Table 1.** Table of Notations

*Symbol/abbreviation*	*Definition*
*F*	Fragment length (in base-pairs; bp)
*T*	Transcript length (in bp)
***FP***(*T*,*F*)	Number of unique fragmentation patterns for fragment length *F* on transcript length *T*, where a fragmentation pattern refers to a unique configuration of exhaustive fragment placement
SP	Fragment starting-point
*R*	Read length (in bp)
|	Concatenation of vectors
***C***	General coverage vector for a fragment with length *F* may represent either the SP coverage, the read coverage, or the fragment coverage
***N****(T,F,C)*	Expected coverage profile from all unique fragmentation patterns for *F* on *T*, depending on the coverage vector ***C*** for each fragment (see previous row)
***SPP***	Starting-point profile; expected coverage profile ***N*** obtained with the starting-point coverage vector ***C***
***FCP***	Fragment coverage profile; expected coverage profile ***N*** obtained with the fragment coverage vector ***C***
***RCP***	Read coverage profile; expected coverage profile ***N*** obtained with the read coverage vector ***C***
*F_k_*	Fragment length (in bp) for the *k*th fragment length in a range of fragment lengths
*W(F_j_)*	Weight for the contribution of the *j*th fragment length, *F_j_*, to the expected coverage profile
***N_w_****(T,F_j__=__1,…, k_,C)*	Expected coverage profile from all unique fragmentation patterns for a range of fragment lengths *F_j_*_=__1,…, *k*_ on transcript *T*, with fragment-specific weights multiplied to the fragment coverage vector ***C***
***E***	Empirical cumulative distribution function (computed from ***N****)*

The observation that fragmentation patterns of shorter transcripts reoccur within longer transcripts (Fig. 2, bordered orange boxes) naturally led us to a recursion to compute the pattern space. In exhaustive fragmentation, the left-most fragment placed on a transcript may not be more than *F* base-pairs (bp) away from the start of the transcript. As such, the fragmentation patterns of the pattern space can be divided into *F* parts, where fragmentation patterns of each part have the left-most fragment beginning on the same distinct position and the pattern space for a shorter transcript on the remaining length. The number of fragmentation patterns (*FP*) in the pattern space for a given *T* and *F*, is the sum over the *F* parts, of the number of unique fragmentation patterns that exist in the pattern space for the remaining length of the transcript, after placement of the first fragment:

\begin{align*}
FP ( T , F ) = \mathop \sum \limits_{i = 0}^{F - 1} {FP ( T - ( F + i ) ,\ F ) } , \quad T > F \tag{1}
\end{align*}

where $$FP ( l,F ) = 1 , \quad l = 0 , \ldots \,, F$$

$$ \qquad FP ( l,F ) = 0 , \quad l < 0$$.

We assume that in unbiased fragmentation, every fragmentation pattern is equally likely. Therefore, for each position along the transcript, we sum the coverage contributed from the fragments observed in the pattern space to obtain the expected coverage profile (***N***). To compute ***N*** using a similar recursion as in Equation 1, we introduce a general coverage vector (***C***) that represents a single fragment (see Equation 2). ***C*** is of length *F* and may be modified to represent the fragment SP, fragment coverage, or read coverage. An SP refers to the left-most position of a fragment placed (boxes shaded red in Fig. 2), the fragment coverage is coverage of all bases within *F*, and the read coverage is coverage of the bases within *R* on each end of a fragment. We make no distinction between the coverage obtained from single- or paired-end sequencing (Fig. 1B) because we assume both ends of the fragment are equally likely to be sequenced. ***C*** is concatenated end-to-end with ***N*** of the pattern space of a shorter transcript of the remaining length required (concatenation depicted as “l” in Equation 2) to obtain a final vector of the same length as the transcript.

The general formula for the coverage profile ***N*** is


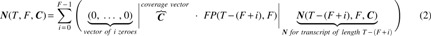


where $${ \textbf{\textit{N}}} ( l,F , { \textbf{\textit{C}}} ) = ( \underbrace {0 , \ldots , 0}_{vector \ of \ l \ zeroes} ) , \quad l = 0 , \ldots\, , F - 1$$

The coverage vector ***C*** can be replaced as follows:

${ \textbf{\textit{C}}} = ( 1 , \underbrace {0 , \ldots, 0}_{F - 1 \ zeroes} )$ for the starting-point profile (***SPP***)

${ \textbf{\textit{C}}} = ( \underbrace {1 , \ldots, 1}_{F \ ones} )$ for the fragment coverage profile (***FCP***)

${ \textbf{\textit{C}}} = ( \underbrace {1 , \ldots, 1}_{R \ ones}, \ \underbrace {0 , \ldots, 0}_{F - R \ zeroes} ) + ( \underbrace {0 , \ldots, 0}_{F - R \ zeroes}, \ \underbrace {1 , \ldots, 1 ) }_{R \ ones}$ for the read coverage profile (***RCP***).

This allows one to generate any of the desired profiles, that is, ***SPP***, ***FCP***, or ***RCP***, from the general recursion simply by specifying the relevant ***C***.

### 2.2. Fragment length to transcript length ratios influence coverage profiles

**FIG. 3.**
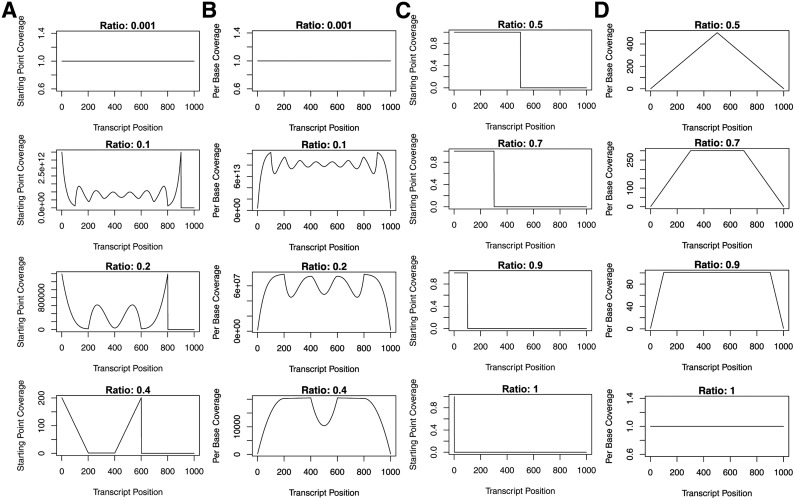
Fragment length to transcript length ratios influence variability of coverage along the transcript. The expected SPPs **(A)** and FCPs **(B)** obtained from the enumerated pattern space for placement of fragments of lengths 1, 100, 200, and 400 (top to bottom) bases long on a transcript of 1000 bases long. The expected ***SPP***s **(C)** and ***FCP***s **(D)** obtained from the enumerated pattern space for placement of fragments of lengths 500, 700, 900, and 1000 (top to bottom) bases long on a transcript of 1000 bases long. ***SPP***s, starting-point profiles; FCPs, fragment coverage profiles.

Figure 3 shows the ***SPP***s (Fig. 3A,C) and the ***FCP***s (Fig. 3B,D) for different *F* to *T* ratios, where *T* = 1000. Uniform coverage is observed for the smallest *F* to *T* ratio, 1/*T*, when *F* is 1 bp (Fig. 3A,B, top-most panel). However, beyond this, the smaller the ratio, the greater the number of peaks observed in the ***SPP*** (Fig. 3A) and ***FCP*** (Fig. 3B). For *F* to *T* ratios greater than 0.5, the ***SPP*** is uniform for all potential SPs (Fig. 3C), whereas the ***FCP*** has a single peak or plateau (Fig. 3D). The closer the ratio is to 1, the broader the plateau and the closer the ***FCP*** is to uniformity. A uniform ***FCP*** is also observed when *F* is equal to *T* (Fig. 3D, bottom-most panel).

### 2.3. Read length to fragment length ratios influence the read coverage profile

**FIG. 4.**
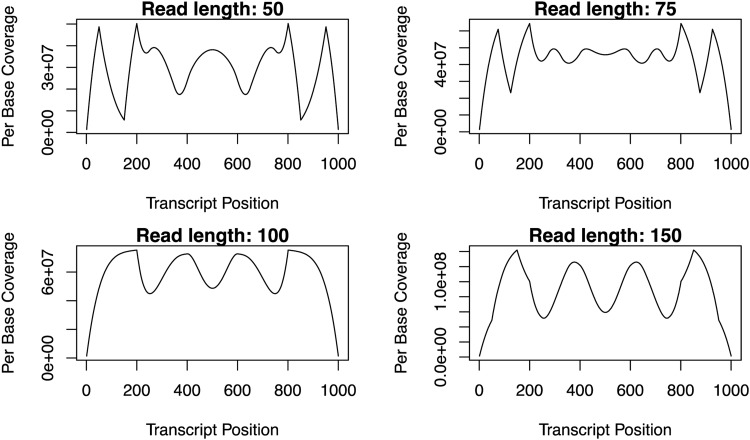
The expected read coverage profiles obtained from the enumerated pattern space for placement of fragments of length 200 bases long on a transcript of 1000 bases long and where the read lengths are 50, 75, 100, and 150 bases long.

When we distinguished *R* from *F* in our model, we find that the smaller the ratio of *R* to *F*, the more pronounced the differences between peaks and valleys of the ***RCP*** (Fig. 4, top row). This is due to a larger portion of each fragment not being sequenced. When the *R* to *F* ratio is 0.5, the full fragment is sequenced and, therefore, the ***RCP*** is the same as the ***FCP*** (Fig. 4, read length 100). Increasing the ratio of *R* to *F* beyond 0.5 (i.e., overlap of forward and reverse reads) also results in more distinct peaks and valleys of the ***RCP*** (Fig. 4, read length 150) compared with the profile where the *R* to *F* ratio is 0.5.

### 2.4. Towards more realistic assumptions

#### 2.4.1. Including a range of fragment lengths

**FIG. 5.**
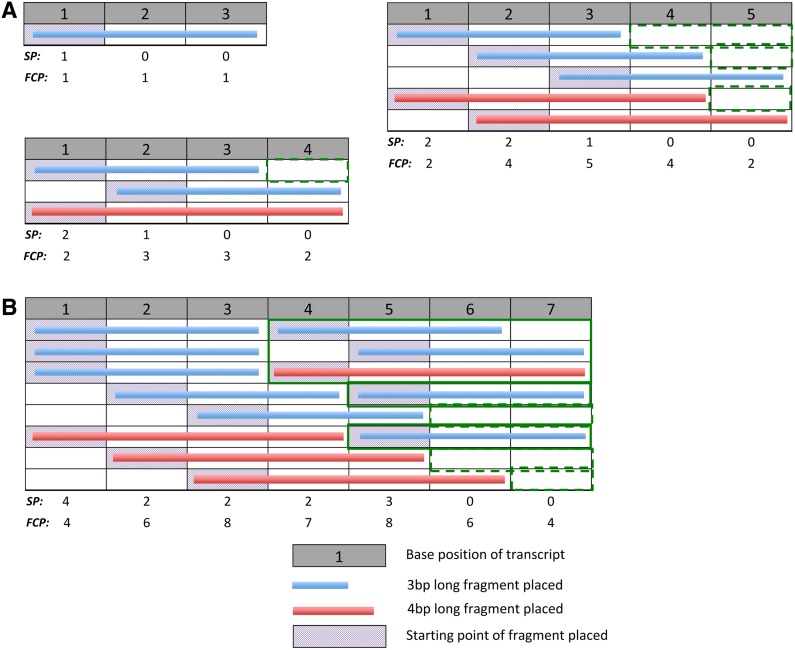
The fragment placement pattern space for various transcript lengths. **(A)** Fragments of 3 and 4 bases long placed on transcripts of lengths 3–5 bases. **(B)** Fragments of 3 and 4 bases long placed on a transcript of 10 bases long. Each row represents a unique fragmentation pattern, where fragments are placed till remaining positions on the transcript permit no further placement of a fragment. The computed ***SPP*** and ***FCP*** are, respectively, the sum of fragment starting-points (boxes shaded red) and the sum of fragments covering each position, and are shown under each pattern space. Sections that have been bordered green show pattern spaces of shorter transcripts found in longer transcripts. Dashed green borders show the pattern spaces for transcripts shorter than the fragment length.

In the experimental fragmentation process, a range of fragment lengths are produced. We, therefore, updated the model to enumerate all unique ways to place a range of fragment lengths, ***F_1_*** to ***F_K_***, on a transcript, length *T*. We illustrate this with simple examples for *F*_1_ = 3 and *F*_2_ = 4 (Fig. 5). The recursion iterates over the range of fragment lengths for placement of the first fragment and similarly uses the pattern spaces of shorter transcripts for the rest of the transcript (Fig. 5, bordered green boxes). One obtains the general formula to calculate the number of fragmentation patterns for a transcript of length *T* and a range of fragments of length *F*_1_ to *F*_K_ (where *F*_1_ < *F*_2_ < … < *F*_k_) as

\begin{align*}
FP ( T , {F_{j = 1 , \ldots, k}} ) = \mathop \sum \limits_{i = 0}^{{F_1} - 1} { \mathop \sum \limits_{j = 1}^k {FP ( T - ( {F_j} + i ) ,\ {F_{j = 1 , \ldots, k}} ) , \quad T > {F_1},} } \tag{3}
\end{align*}

where $$FP ( l, {F_{j = 1 , \ldots , k}} ) = 1 , \ l = 0 , \ldots , {F_1}$$

$$ \qquad FP ( l,{F_{j = 1 , \ldots , k}} ) = 0 , \ \ l < 0$$.

The general formula for the coverage profile, ***N***, for a transcript of length *T* and a range of fragments of length *F*_1_ to *F*_K_ is 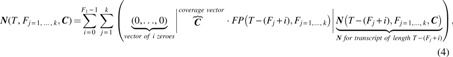


where $$\textbf{\textit{N}} \left( {l,{F_{j = 1 , \ldots, k}}, \textbf{\textit{C}}} \right) = ( 0 , \ldots, 0 ) , \quad l= 0 , \ldots, {F_1} - 1$$

and ***C*** is taken as the following:

$( 1 , \underbrace {0 , \ldots, 0}_{{F_j} - 1 \ zeroes} )$ for the ***SPP***

$( \underbrace {1 , \ldots, 1}_{{F_j} \ ones} )$ for the ***FCP***

$( \underbrace {1 , \ldots, 1}_{R \ ones}, \ \underbrace {0 , \ldots, 0}_{{F_j} - R \ zeroes} ) + ( \underbrace {0 , \ldots, 0}_{{F_j} - R \ zeroes}, \ \underbrace {1 , \ldots, 1}_{R \ ones} )$ for the ***RCP***.

#### 2.4.2. Imposing an experimentally derived fragment length distribution on the pattern space

In an RNA-seq experiment, fragment lengths occur at different frequencies. The fragment length distribution can be measured before sequencing of the input DNA (Panaro et al., 2000), but this information is typically not provided with RNA-seq data sets. We downloaded an RNA-seq library (SRR897347) of synthetic sequences (ERCC) from the Sequencing Quality Control (SEQC) project (SEQC-Consortium, 2014) and mapped the reads to a reference of ERCC sequences (Rosenbloom et al., 2013) with NextGenMap (Sedlazeck et al., 2013) using parameters—min_identity 0.9 and min_residues 1—such that the full length of read is mapped (note that 18.57% of reads remained unmapped). An empirical distribution of fragment lengths was determined from the difference between genomic positions of two extreme ends of mapped read pairs for the sequence ERCC-00002. We used this empirical distribution to linearly interpolate unobserved fragment lengths within the range of observed fragment lengths using *na.approx* (Zeileis and Grothendieck, 2005) in *R*. Next, the weight, ***W***, for each fragment length was calculated as the ratio of relative observed frequency of fragment length to the relative expected frequency in the pattern space of the model:

\begin{align*}
W ( { F_j } ) = { \frac { Relative \ observed \ frequency \ of \ { F_j } } { Relative \ expected \ frequency \ of \ { F_j } } } = { \frac { Data } { Model } } 
\end{align*}

*W(F_j_)*: Weight for fragment length *F_j_*

We modify Equation 4 to obtain the general formula, ***N_w_***, for coverage profiles obtained in the weighted pattern space: 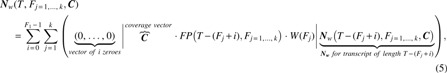


where $${\textbf{\textit{N}}_w} ( l,{F_{j = 1 , \ldots, k}},\textbf{\textit{C}} ) = ( 0 , \ldots, 0 ) , \ l = 0 , \ldots, {F_1} - 1$$.

### 2.5. Fragment length range influences the coverage profile

**FIG. 6.**
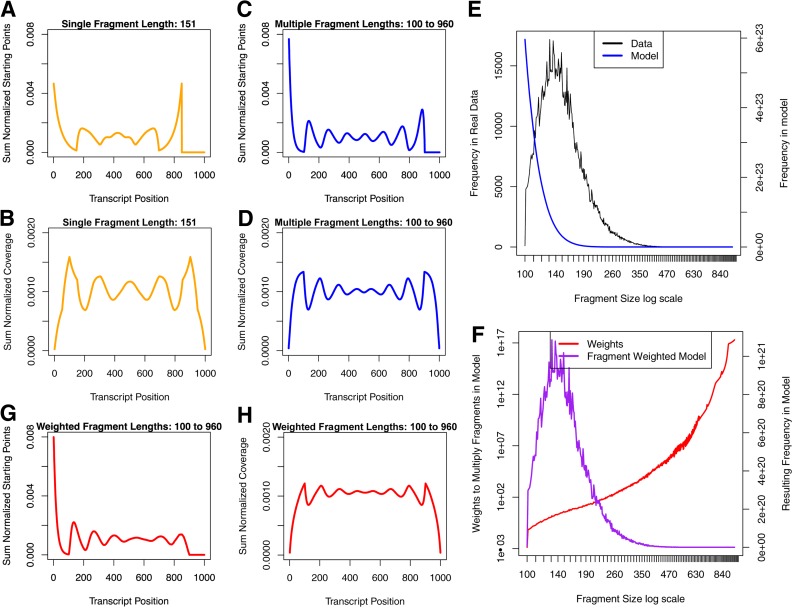
The influence of fragment size range and distribution on the ***SPP*** and RCP for a transcript of length 1000 bases and read length of 100 bases. The ***SPP*** **(A)** and RCP **(B)** in the pattern space with a single fragment size of 151 bases long. The ***SPP*** **(C)** and RCP **(D)** in the pattern space with fragment sizes from 100 to 960 bases long. **(E)** An empirical fragment size distribution (black, left y-axis) of 1,260,673 fragments, calculated from the insert sizes of paired-end reads mapping to a transcript, synthetic spike-in ERCC-00002, from a single lane (SRR897347) of the Sequencing Quality Control (SEQC) project (SEQC/MAQC-III Consortium, 2014), and the fragment size distribution in the enumerated pattern space with fragment sizes 100 to 960 bases long (blue, right y-axis). **(F)** The weights multiplied to each fragment in the pattern space (red, left y-axis) were calculated by the ratio of relative observed frequency of the fragment size in the data to the relative expected frequency in the pattern space. This resulted in a transformed fragment size distribution of the pattern space (purple, right y-axis). The ***SPP*** **(G)** and the RCP **(H)** in the weighted pattern space with fragment sizes from 100 to 960 bases long. Note: All profiles have been normalized to have a sum of 1. RCP, read coverage profile.

We compared the results of enumerating the pattern space using a single fragment length (151 bp—the median fragment length mapped to ERCC-00002) to the results of enumerating the pattern space using a range of fragment lengths (100–960 bp—the range for fragment lengths mapped to ERCC-00002) with *T* = 1000 bp with *R* = 100 bp. The ***SPP*** (Fig. 6A) and ***RCP*** (Fig. 6B) in the pattern space with a single fragment length contain less peaks than the ***SPP*** (Fig. 6C) and ***RCP*** (Fig. 6D) of the pattern space with a range of fragment lengths, due to the inclusion of fragment lengths smaller than 151 bp. In addition, since fragments with lengths larger than 0.5 of the transcript length start within the first half of the transcript, there is a clear asymmetry in the ***SPP*** of the pattern space having a range of fragment lengths (Fig. 6C). Less pronounced differences between peaks and valleys of the ***RCP*** were also observed (Fig. 6D).

### 2.6. Fragment length distribution influences the coverage profile

The fragment length distribution from the enumerated pattern space is L shaped, whereas the empirical fragment length distribution that was inferred from reads mapping to the sequence ERCC-00002 is bell shaped (Fig. 6E). Imposing the empirical fragment length distribution on the pattern space using weights (Fig. 6F) results in less pronounced differences between peaks and valleys in the second half of the ***SPP*** (Fig. 6G). As the weights of long fragments are greater than weights of short fragments (Fig. 6F, red line), the SPs of shorter fragments contribute less to the ***SPP***. This is only evident in the second half of the transcript, where there are no SPs of long fragments. Also, the variability across the ***RCP*** is greatly reduced in the weighted pattern space (Fig. 6H).

### 2.7. Simulating the RNA-Seq experiment: sampling of the pattern space

**FIG. 7.**
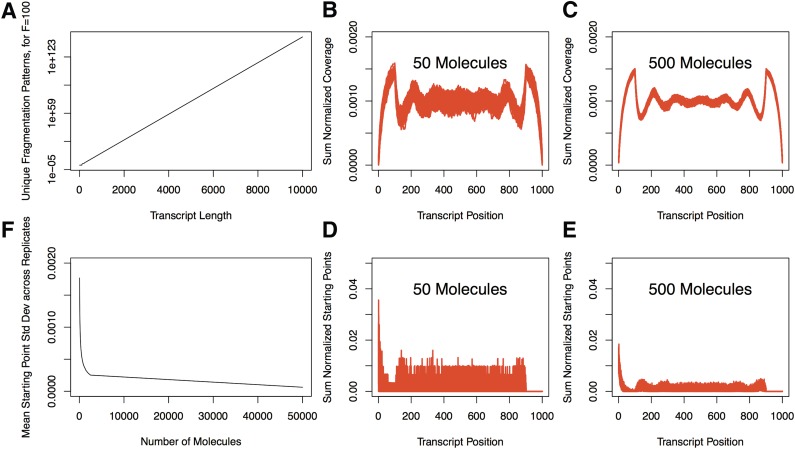
Sampling from the pattern space. **(A)** The number of unique fragmentation patterns for fragment length 100 and increasing transcript lengths. The coverage profiles of 100 simulations generated with 50 original molecules **(B)** and 500 original molecules **(C)**. The starting-point profiles of 100 simulations generated with 50 original molecules **(D)** and 500 original molecules **(E)**. **(F)** The mean transcript-position standard deviation of sum-normalized starting-points between the 100 simulations for increasing number of molecules.

*FP* for a specific fragment length grows exponentially with increasing transcript length (Fig. 7A). As the number of copies of a transcript is less than the number required to represent the full pattern space, we “fragmented” a defined number of molecules by exhaustively assigning fragments on the transcript based on the ***SPP*** and the position-specific fragment length distribution of an enumerated pattern space—where *T* = 1000, *R* = 100, and an imposed empirical fragment length distribution obtained from an RNA-seq library SRR896983 (SEQC-Consortium 2014) (note that 12.07% of reads remained unmapped). Simulated “fragmentation” was performed for 43 values between 50 molecules and 50,000 molecules. For each set of fragmented molecules, we uniformly amplified all fragments by 2^15 (PCR-Cycles)^ = 32,768 times to simulate perfectly efficient PCR amplification and then simulated sequencing by sampling fragments to finally generate an ***SPP***. Each simulated sequencing experiment was done with 100 repetitions. In general, we found that the smaller the number of molecules we started with, the greater the variability we observed between the ***RCP***s (50 molecules vs. 500 molecules for Fig. 7B,C) and ***SPP***s within 100 repetitions (50 molecules vs. 500 molecules for Fig. 7D,E; mean transcript position standard deviation between repetitions for all values of number of molecules evaluated for Fig. 7F).

### 2.8. Estimating the original number of molecules

**FIG. 8.**
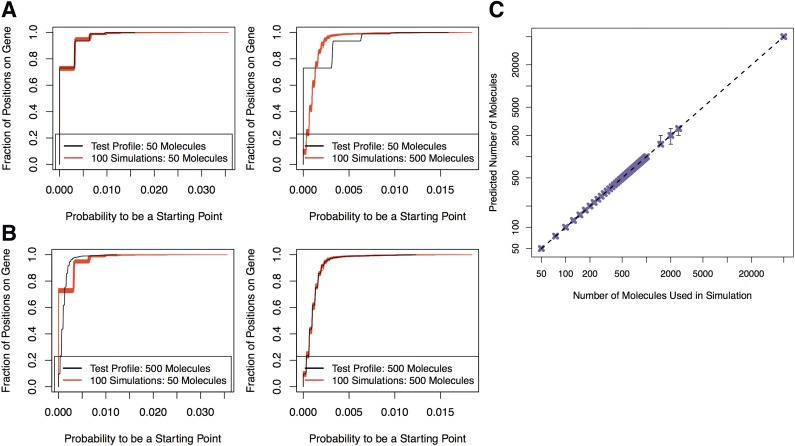
Estimating the number of molecules that were sequenced. **(A)** Comparing the ECDF of a test profile generated with 50 molecules (black) and the ECDFs of 100 repetitions generated from 50 molecules (left) or 500 molecules (right). **(B)** Comparing the ECDF of a test profile generated with 500 molecules (black) and the ECDFs of 100 repetitions generated from 50 molecules (left) or 500 molecules (right). **(C)** The predicted number of molecules compared with the original number of molecules that were used in the simulation. The identity line is included (dashed, black). Error bars show the maximum and minimum number of molecules that were predicted of 100 repetitions of each prediction procedure. ECDF, empirical cumulative distribution function.

With the same procedure described in the previous section we generated an additional test profile for each of the 43 values evaluated between 50 molecules and 50,000 molecules and compared the test profiles with all 43 sets of the 100 repetitions. To make comparisons between profiles, the ***SPP***s were first converted into empirical cumulative distribution functions (ECDFs) (Fig. 8A,B), we then computed the distance between the ECDF of a test profile to the ECDF for the set of 100 repetitions per number of molecules.

The distance between two ECDFs, ***E***_*m*_ and ***E***_*d*_, is as follows:

\begin{align*}
= \mathop \sum \limits_{i = 1}^{n - 1} \bigg (({E_m} ( {x_i}  ) -
{E_d} ( {x_i})) ^{2} \ ( {x_{i + 1}} - {x_i}) \bigg ) ,
\end{align*}

where ${ \textbf{\textit{x}}} = ( {x_1}, \ldots , {x_n} )$ is the unique list of observed values, in increasing order, of sum-normalized SP counts in the ECDFs being compared.

Based on the set with the smallest distance to the test profile ECDF, we predicted the original number of molecules the test profile was generated with. We found that the ***SPP***s were distinct enough between ECDFs over the range of molecules evaluated to predict the correct original number of molecules (Fig. 8C). The accuracy was consistent in 100 repetitions of each prediction procedure.

## 3. Discussion

This article explored a model for the expected distribution of fragment SPs for unbiased coverage across a single transcript in RNA-seq. We model the products of fragmentation by enumerating all possible unique ways to obtain the maximum number of fragments of a desired length from a transcript. This is pertinent to recent RNA-seq projects of smaller amounts of starting material (Adiconis et al., 2011; Saliba et al., 2014), where the majority of the fragments from each molecule are sampled. Read coverage in RNA-seq has so far been nonuniform; for instance, read counts modeled as Poisson variables with constant rates along each transcript fit data poorly (Li et al., 2010) and the majority of transcripts evaluated by Hower et al. (2012) were found not to have homogeneity of starting-point and fragment length distribution along the transcript. Although nonuniformity in coverage is often attributed to biases in the experiment (Wu et al., 2011; Benjamini and Speed, 2012), we found that the expected unbiased coverage profile is dependent on the fragment length to transcript length ratio, read length to fragment length ratio, and fragment length distribution. Our results have implications on the methods that assume the expected coverage is uniform (Jiang and Wong, 2009; Li et al., 2010; Hower et al., 2012). Based on our analysis, some variability in the SPs is expected and thus investigations of nonuniformity should be aimed at positions with observations outside these expected values.

As opposed to the Poisson (Jiang and Wong, 2009) and negative binomial (Miller et al., 2011) models that provide a numerical measure to capture the distribution of the data, the pattern space model is based on a spatial representation of the products of fragmentation. Identical configurations have also been described for a discrete car-parking problem (Texter, 1989), but unlike the car-parking problem, we assume all patterns have the same probability of occurring. We find this results in greater packing densities in the exhaustive fragmentation pattern space than the discrete car-parking problem (data not shown). In our analysis, we inferred the fragment length distribution of a transcript based on mapped reads, and this may not be the fragment length distribution after fragmentation. Nonetheless, the model provides insights into how the fragment length distribution influences the variability of the SP distribution.

In future, we plan to evaluate whether the trends we observed with our proposed pattern space model occur in experimental data sets that were prepared from both limited number of molecules and exhaustive sequencing. Given that we were able to accurately predict the number of molecules we used in RNA-seq simulations, it would also be of great interest to evaluate the potential to determine the original number of molecules in empirical RNA-seq data sets.
